# Physiological and Transcriptomic Analysis Reveals Distorted Ion Homeostasis and Responses in the Freshwater Plant *Spirodela polyrhiza* L. under Salt Stress

**DOI:** 10.3390/genes10100743

**Published:** 2019-09-24

**Authors:** Lili Fu, Zehong Ding, Xuepiao Sun, Jiaming Zhang

**Affiliations:** Institute of Tropical Bioscience and Biotechnology, MOA Key Laboratory of Tropical Crops Biology and Genetic Resources, Hainan Academy of Tropical Agricultural Resource, Hainan Bioenergy Center, Chinese Academy of Tropical Agricultural Sciences, Xueyuan Road 4, Haikou 571101, China; fulili@itbb.org.cn (L.F.); dingzehong@itbb.org.cn (Z.D.); sunxuepiao@itbb.org.cn (X.S.)

**Keywords:** salt stress, freshwater plants, duckweed, *Spirodela polyrhiza*, RNA-seq analysis, expression regulation, ion homeostasis

## Abstract

Duckweeds are a family of freshwater angiosperms with morphology reduced to fronds and propagation by vegetative budding. Unlike other angiosperm plants such as *Arabidopsis* and rice that have physical barriers between their photosynthetic organs and soils, the photosynthetic organs of duckweeds face directly to their nutrient suppliers (waters), therefore, their responses to salinity may be distinct. In this research, we found that the duckweed *Spirodela polyrhiza* L. accumulated high content of sodium and reduced potassium and calcium contents in large amounts under salt stress. Fresh weight, Rubisco and AGPase activities, and starch content were significantly decreaseded in the first day but recovered gradually in the following days and accumulated more starch than control from Day 3 to Day 5 when treated with 100 mM and 150 mM NaCl. A total of 2156 differentially expressed genes were identified. Overall, the genes related to ethylene metabolism, major CHO degradation, lipid degradation, N-metabolism, secondary metabolism of flavonoids, and abiotic stress were significantly increased, while those involved in cell cycle and organization, cell wall, mitochondrial electron transport of ATP synthesis, light reaction of photosynthesis, auxin metabolism, and tetrapyrrole synthesis were greatly inhibited. Moreover, salt stress also significantly influenced the expression of transcription factors that are mainly involved in abiotic stress and cell differentiation. However, most of the osmosensing calcium antiporters (OSCA) and the potassium inward channels were downregulated, Na^+^/H^+^ antiporters (SOS1 and NHX) and a Na^+^/Ca^2+^ exchanger were slightly upregulated, but most of them did not respond significantly to salt stress. These results indicated that the ion homeostasis was strongly disturbed. Finally, the shared and distinct regulatory networks of salt stress responses between duckweeds and other plants were intensively discussed. Taken together, these findings provide novel insights into the underlying mechanisms of salt stress response in duckweeds, and can be served as a useful foundation for salt tolerance improvement of duckweeds for the application in salinity conditions.

## 1. Introduction

Duckweeds belong to the Lemnaceae family and include five genera of *Spirodela*, *Landoltia*, *Lemna*, *Wolffiella*, and *Wolffia* with a total of 37 species [1]. In recent years, duckweeds have attracted more and more attention and are recognized as a model for aquatic plants, due to their ideal characteristics (e.g., fast biomass accumulation, high starch content, and high rate of nutrient intake) for livestock feed, wastewater treatment, and bio-ethanol production [2,3]. However, the morphology of duckweeds is highly reduced to only fronds with a few roots or no roots at all [4]. The reduced morphology results in loss of physical barriers between their photosynthetic organs and nutrient suppliers unlike other angiosperms (e.g., *Arabidopsis* and rice). This may explain why duckweeds are highly sensitive to salt stress, a major known factor that causes cellular damage and affects plant growth and development. To date, many efforts have been made to characterize the performances of diverse duckweed species in response to salt. In *Spirodela polyrhiza* L., NaCl treatment significantly reduced photosynthetic pigment accumulation, inhibited plant growth, and simultaneously enhanced malondialdehyde (MDA) and hydrogen peroxide (H_2_O_2_) contents in plant tissues [5,6]. Reactive oxygen species (ROS) scavenging system was subsequently activated to protect against the provoked oxidative damage, since the activities of many antioxidative enzymes including superoxide dismutase (SOD), peroxidase (POD), catalase (CAT), and ascorbate peroxidase (APX) were significantly increased [5,6]. In *Lemna minor* L., salt stress strongly reduced the removal capacity of nitrogen (N) and phosphorus (P) from water. Moreover, higher salt stress with longer cultivation period exerted more injury to duckweed and greater inhibition of N and P removal [7]. In *Lemna gibba* L., salt treatment significantly inhibited photosystems I (PSI) and PSII activities, and decreased the overall activity of electron transport chain while stimulating ROS production. Further investigation revealed that ROS production was highly correlated to the maximal quantum yield of PSII [8]. Notably, as many as 34 duckweed strains from all five genera and amongst 13 species were recently selected to quantify the natural variance of traits (including dry weight, growth rate, and starch content) under saline condition, and large ranges of interspecific and intraspecific variations in salt tolerance were revealed [9]. Overall, these findings focused mainly on physiological changes, and provide valuable foundations for further characterization of salt stress responses in duckweeds, however, the regulatory mechanism underlying the salt stress remains largely elusive in duckweeds. 

RNA-seq is a common approach to quantify gene expression during plant development and upon various stress stimuli, and has been widely applied to identify specific trait-associated genes and regulatory networks in plants [10,11]. It is also becoming a useful tool for genome-wide investigation in duckweeds after major progress has been made in genome and transcriptome sequencing [3,12,13,14]. For examples, RNA-seq was employed to identify differentially expressed genes associated with nutrient starvation [15], uniconazole induction [16,17], and cadmium treatment [18] in *Landoltia punctata* (G. Meyer) Les & Crawford; ABA treatment [19] in *S. polyrhiza*; NH_4_^+^ toxicity [20] and ionising radiation [21] in *L. minor*; and nitrogen starvation [22] in *L. aequinoctialis* Welw. However, to the best of our knowledge, currently no RNA-seq analyses concerning salt stress have been performed in duckweeds. Therefore, it is very necessary to complement this gap to improve our understanding of the mechanisms of salt stress in this species. 

In this study, *S. polyrhiza* (strain No. 7498), which is easy to culture and handle and has high growth rate in laboratory condition, was selected to investigate the physiological changes in response to salt stress with three different concentrations. Later, RNA-seq was used to survey the dynamic changes of gene expression at different time points of salt treatment. The results will provide novel insights into the underlying mechanisms of salt stress response, and can be served as a useful foundation for improving salt tolerance of duckweeds. 

## 2. Results

### 2.1. Physiological Changes of S. Polyrhiza under Salt Stress

**Figure 1:**
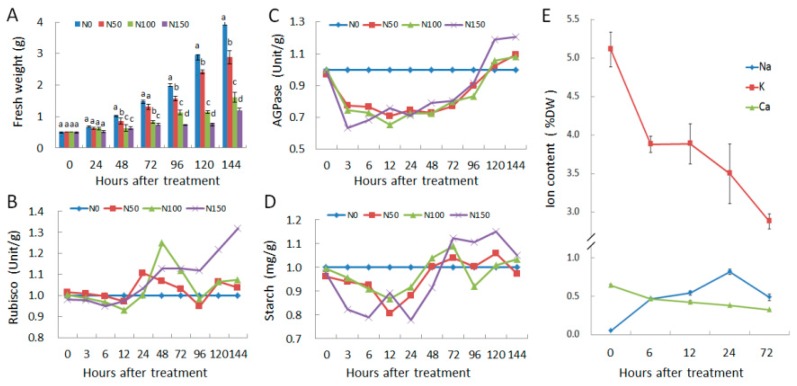
Physiological responses of duckweed (*Spirodela polyrhiza*) in response to salt treatments. Fresh weight (**A**), relative Rubisco enzyme activity (**B**), relative AGPase activity (**C**), relative starch content (**D**) and Ion content (**E**) were investigated under four salt concentrations of 0, 50, 100, and 150 mM NaCl (designated as N0, N50, N100, and N150, respectively) across ten time-points (0, 3, 6, 12, 24, 48, 72, 96, 120, and 144 h) within six days. Sodium, potassium, and cassium contents were measured at 0, 6, 12, 24, and 72 h and presented in percent dry weight (%DW). The relative enzyme activities and starch content are normalized to the levels of 0 mM NaCl treatment (controls) at each time-point, so that the controls are shown as a constant 1, and the treatments (50, 100, 150 mM NaCl) are shown as relative levels to the controls. Different letters on columns in Figure 1A indicate significant difference as tested using one-way ANOVA at 5% significance level.

In order to reveal the physiological changes of *S. polyrhiza* in response to salt stress, four traits, including fresh weight, Rubisco enzyme activity, AGPase activity, and starch content were determined under four salt concentrations (0, 50, 100, and 150 mM NaCl, designated as N0, N50, N100, and N150 respectively hereafter) across ten time-points (0, 3, 6, 12, 24, 48, 72, 96, 120, and 144 h) in *S. polyrhiza* (Figure 1). To eliminate the influence of unexpected environments, e.g., circadian rhythms on Rubisco enzyme activity and other physiological traits, 0 mM NaCl treatment (N0) was used as controls at all time-points.

Figure 1A shows that fresh weight of the control (N0) was almost doubled every two days, and increased by 7.8 folds in total by the end of the experiment (Day 6). The salt-treated plants showed reduced growth in a dose and temporal-dependent manner, and the reduction was more obvious as treatments went on and/or NaCl concentration was increased (Figure 1A). The fresh weights were reduced by 7.4%, 8.2%, and 22.2% in the first day for N50, N100, and N150, respectively, and were reduced by 26.3%, 58.8%, and 69.7% for N50, N100, and N150, respectively by the end of the experiment (Day 6). The fronds of N50 were morphologically as healthy as control plants (N0) until the end of the experiment, and no obvious morphological changes were observed in fronds of N100 and N150 after treated for 4 days. However, at Day 5, some fronds of N100 and N150 showed discoloration (approximately 4% and 6% fronds for N100 and N150, respectively), and many fronds of N150 turned yellow at Day 7 (Supplementary Figure S1), which is similar to previous reports [5,6].

Ribulose-1,5-bisphosphate carboxylase/oxygenase (Rubisco) protein levels are constant in some plants [23], but its activity is highly regulated in response to fluctuations in the environment [24]. Results indicate that rubisco activities in three treatments all declined in the first 12 h and increased at 24 h, then three treatments showed different patterns (Figure 1B). The N50 and N10 downregulated rubisco activity in the next two days, but upregulated again. N150, however, upregulated rubisco activity steadily after 24 h until the end of the experiment. 

The reaction catalyzed by AGPase is considered as the first and key step in starch biosynthesis [25]. AGPase activity responded rapidly to NaCl treatment, and declined sharply at 3 h (Figure 1C). N150 plants responded more rapidly and reached the lowest point at 3 h, while N50 and N100 reached the lowest point at 12 h. Then AGPase activity increased gradually, and reached levels even higher than the control at 120 h and 144 h (Figure 1C). Similarly, the starch content decreased in all three treatments in the first few hours (Figure 1D), and N150 plants responded most rapidly and reached the lowest point at 6 h, while N50 and N100 plants reached the lowest point at 12 h. However, starch content increased after 72 h, especially for the N150 plants, which accumulated more starch than N0 plants from 72 h to 120 h. Coincidently, final starch content was reported to increase under salt stress in many duckweed species [9]. However, the decrease in the beginning of the stress was not reported.

In order to reveal the changes in ion status of *S. polyrhiza* in response to salt stress, sodium, potassium, and calcium contents were measured under 100 mM NaCl treatment at time points of 0, 6, 12, 24, and 72 h after treatment. Results indicated that the sodium content before salt stress was quite low (0.05 %DW), however, sodium content increased to 0.47 %DW after 6 h treatment, and reached the highest level (0.82 %DW, 16.4 folds increase) at 24 h, and then declined to 0.49 %DW at 72 h (Figure 1E). Consequently, potassium and calcium ion contents all decreased continuously, with the most rapid decline at 6 h. At the end of the experiments (72 h), the potassium content decreased from 5.11 %DW to 2.88 %DW, and calcium content decreased from 0.65% to 0.33 %DW (Figure 1E). These results indicated that 100 mM salt treatment strongly disturbed the ion homeostasis in *S. polyrhiza*.

Taken together, Rubisco enzyme activity, AGPase activity, and potassium and calcium intake in *S. polyrhiza* were significantly inhibited by salt stress, and consistent changes occurred during the early periods (e.g., from 0 h to 24 h) under 50 and 100 mM NaCl treatments. Thus, the samples of *S. polyrhiza* were subsequently collected at 0, 6, 12, and 24 h, respectively, under 100 mM NaCl treatment, and subjected to RNA-seq sequencing to further explore the changes of gene expression at the transcriptional level.

### 2.2. Transcriptome Changes in Response to Salt Stress

In total, 1002 million raw reads with 150-bp in length were obtained by pair-end Illumina sequencing. After removing adapters and low-quality reads, about 868 million (86.6 %) clean reads were retained and subsequently mapped to the *S.* *polyrhiza* reference genome (phytozome version 2). The expressed genes were arbitrarily identified by a threshold cutoff, FPKM (Fragments Per Kilobase of exon per Million fragments mapped) > 10, to minimize the false positive rate. In total, 11,012 expressed genes, which is equivalent to about three-fifths of the annotated genes in the *S. polyrhiza* genome, were identified.

**Figure 2:**
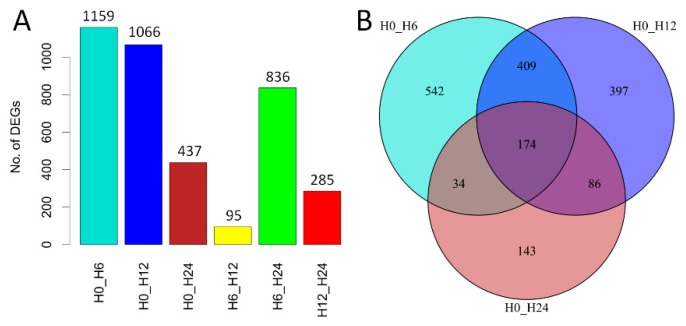
Transcriptome profiling of *Spirodela polyrhiza* responding to salt treatment. (**A**) Number of differentially expressed (DE) genes identified by pairwise comparison; (**B**) Venn diagrams of DE genes identified between 0 h and 6, 12, and 24 h, respectively. H0, H6, H12, and H24 represent the samples collected from 0, 6, 12, and 24 h after salt treatment, respectively.

To demonstrate the gene expression changes triggered by salt stress, differentially expressed (DE) genes were identified by pair-wise comparison (FDR < 0.05 and the absolute value of log_2_FC ≥ 1) of samples collected at different time-points, respectively. There were 1159, 1066, and 437 DE genes identified between 0 h and 6, 12, and 24 h, respectively (Figure 2A). Among which, 542, 397, and 143 DE genes were uniquely identified while only 174 were commonly identified (Figure 2B). In contrast, there were 95, 836, and 285 DE genes identified between 6 h and 12 h, and between these two time-points and 24 h, respectively (Figure 2A). These results indicated that salt stress-triggered more DE genes at the early-stage than at the late-stage of treatment in duckweed. 

### 2.3. Expression Patterns of DE Genes and Functional Enrichment

**Figure 3:**
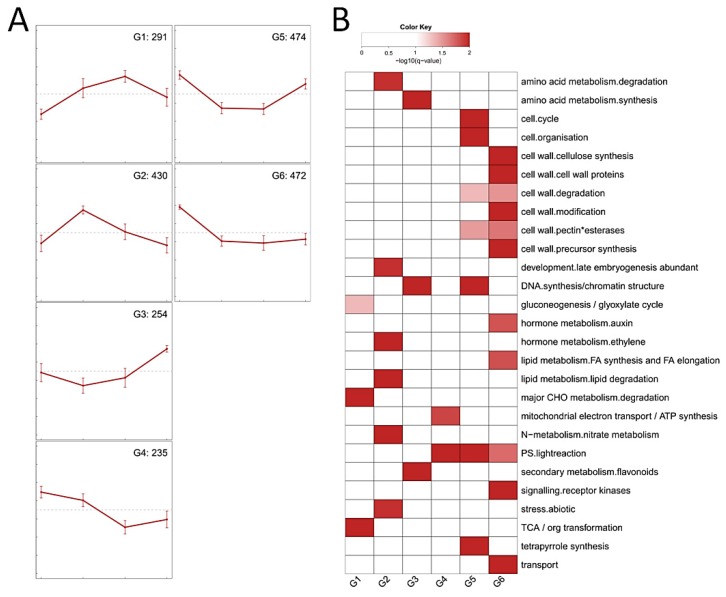
Transcriptional changes of genes involved in salt stress. (**A**) Gene expression patterns of six groups (G1–G6) under salt treatment. The samples (from left to right) are derived from 0, 6, 12, and 24 h after salt treatment, respectively. Error bars represent standard deviation, the numbers of DE genes are indicated in the upper right corner. (**B**) Functional enrichment of genes from each group in (**A**).

In order to characterize the biological functions of genes responding to salt stress, totally 2,156 DE genes (~11% of annotated genes of the reference genome, Table S1) were identified and then subjected to perform hierarchical clustering with Pearson correlation method, resulting in a total of six groups (G1–G6, Figure 3) according to their expression trends. Subsequently, functional enrichment analysis was conducted for each group to determine the common and different biological pathways.

Overall, the expression levels of genes from group G1 to G3 were greatly induced by salt treatment. There were 291 DE genes in group G1, of which the genes were significantly upregulated from 0 h to 12 h but downregulated at 24 h. These genes were enriched in gluconeogenesis, degradation of major CHO metabolism, and TCA transformation. There were 430 DE genes in group G2. These genes were significantly upregulated from 0 h to 6 h but then gradually declined until 24 h (Figure 3A), and they were significantly enriched in degradation of amino acid metabolism, ethylene metabolism, lipid degradation, N-metabolism, and abiotic stress (Figure 3B). There were 254 DE genes in group G3. The expression levels of these genes slightly fluctuated from 0 h to 12 h, but they were drastically increased from 12 h to 24 h. The enriched categories included synthesis of amino acid metabolism, DNA synthesis/chromatin structure, and secondary metabolism of flavonoids (Figure 3B).

Compared with group G1 to G3, the expression levels of genes in group G4 to G6 were greatly downregulated by salt treatment. There were 235 DE genes in group G4. These genes were gradually decreased from 0 h to 12 h and then slightly changed until 24 h, they were significantly enriched in mitochondrial electron transport of ATP synthesis, and light reaction of photosynthesis. There were 474 and 472 DE genes in group G5 and G6, respectively. The gene expression levels of these two groups were all drastically declined from 0 h to 6 h then became flat until 12 h, however, the genes of group G5 were greatly induced from 12 h to 24 h while those of group G6 were still slightly changed (Figure 3A). The enriched categories in G5 included cell cycle, cell organization, DNA synthesis/chromatin structure, light reaction of photosynthesis, and tetrapyrrole synthesis, while those in G6 included many cell wall-related pathways, auxin metabolism, FA synthesis, and FA elongation, light reaction of photosynthesis, signaling of receptor kinases, and transport (Figure 3B). 

To sum up, the DE genes in group G1 to G6 suggested that the major biochemical shifts along time-series were caused by the highly dynamic and coordinated transcriptional changes of *S. polyrhiza* under salt treatment. In the next subsections, the gene expression profile of certain pathways was further examined to demonstrate their responses to salt stress. 

### 2.4. Photosynthesis, Tetrapyrrole Synthesis, and Carbohydrate Metabolism

**Figure 4:**
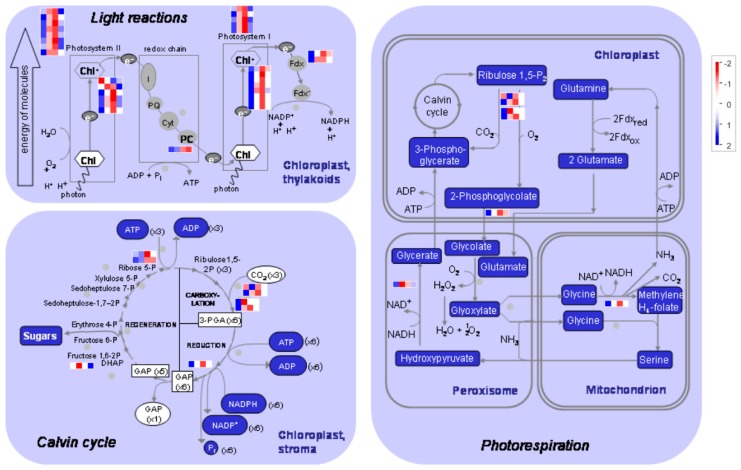
Diagram of DE genes related to photosynthesis pathways. The analysis is performed in the MapMan software. The Fragments Per Kilobase of exon per Million fragments mapped (FPKM) values are transformed by 2-based logarithm and then normalized by row as scaled by the color bar. Each row indicates a single gene, and the cells (from left to right) represent the samples from 0, 6, 12, and 24 h after salt treatment, respectively.

Genes related to photosynthesis pathways were dramatically downregulated by salt stress, especially after 6 h and 12 h of treatment (Figure 4 and Table S2). Most of these genes were involved in light reaction, including PS I (e.g., *LHCA1* to *LHCA4*), PS II (e.g., *LHCB2* to *LHCB6*), and their related polypeptide subunits (e.g., PSAD and PSAF, and PSBP and PSBQ). Consistently, DE genes related to electron carrier and state transition of light reaction were also declined (Table S2). In contrast to light reaction, only a few genes related to calvin cycle (including Rubisco small subunits and Rubisco activase) and photorespiration were changed (Table S2).

Tetrapyrroles play an important role in photosynthesis and respiration. As expected, several genes involved in tetrapyrrole synthesis, including LESION INITIATION 2 (*LIN2*), hydroxymethylbilane synthase (*HEMC*), protochlorophyllide oxidoreductase A (*PORA*), and *PORC*, were greatly downregulated by salt stress (Table S1). 

**Figure 5:**
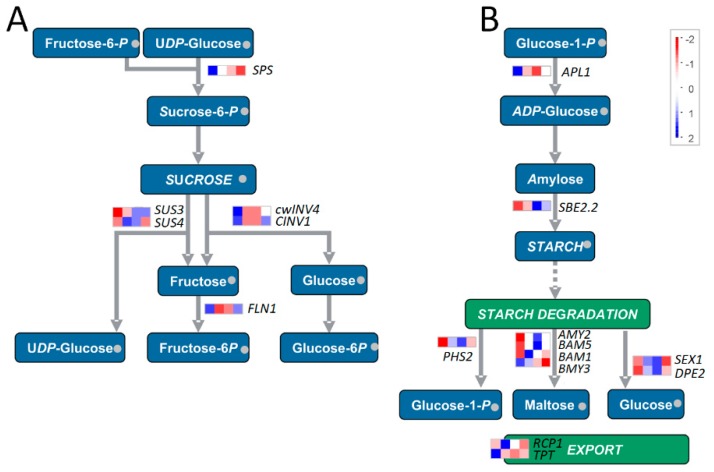
Diagram of DE genes related to sucrose (**A**) and starch (**B**) biosynthesis-degradation pathways. The analysis is performed in the MapMan software. The FPKM values are transformed by 2-based logarithm and then normalized by row as scaled by the color bar. Each row indicates a single gene, and the cells (from left to right) represent the samples from 0, 6, 12, and 24 h after salt treatment, respectively.

Likely, the expression levels of genes involved in starch and sucrose metabolisms were also greatly downregulated by salt. For example, the expression of ADP-glucose pyrophosphorylase (*APL1*) which catalyzes the first and rate-limiting step of starch biosynthesis, and beta-amylase 3 (*BMY3*) and starch transporter (*TPT*) located on the starch-degradation pathway was significantly decreased (Figure 5 and Table S3). Similarly, the expression of sucrose-phosphate synthase (*SPS*) located on the sucrose-synthesis pathway, and cell wall invertase 4 (*cwINV4*), cytosolic invertase 1 (*CINV1*), and fructokinase-like 1 (*FLN1*) located on the sucrose-degradation pathway was also significantly decreased. However, we noted that the expression levels of several genes, including AGPase (*APL2*) and starch branching enzyme (*SBE2*) of starch-synthesis pathway, cytosolic/plastidic alpha-glucan phosphorylase, alpha-amylase, beta-amylase, glucan water dikinase (*SEX1*), and disproportionating enzyme 2 (*DPE2*) of starch-degradation pathway, and sucrose synthases (*SUS3* and *SUS4*) of sucrose-degradation pathway, were greatly induced by salt stress (Figure 5 and Table S3). 

Together, the results suggested that salt stress dramatically downregulated the expression of genes related to photosynthesis and tetrapyrrole synthesis, but bi-directionally changed (either upregulated or downregulated) the expression of genes related to starch and sucrose metabolisms, indicating a possible requirement to maintain the balance between synthesis and degradation of carbohydrate metabolism (e.g., starch and sucrose metabolisms) in response to salt stress.

### 2.5. Response of Cell Wall-Related Genes

**Figure 6:**
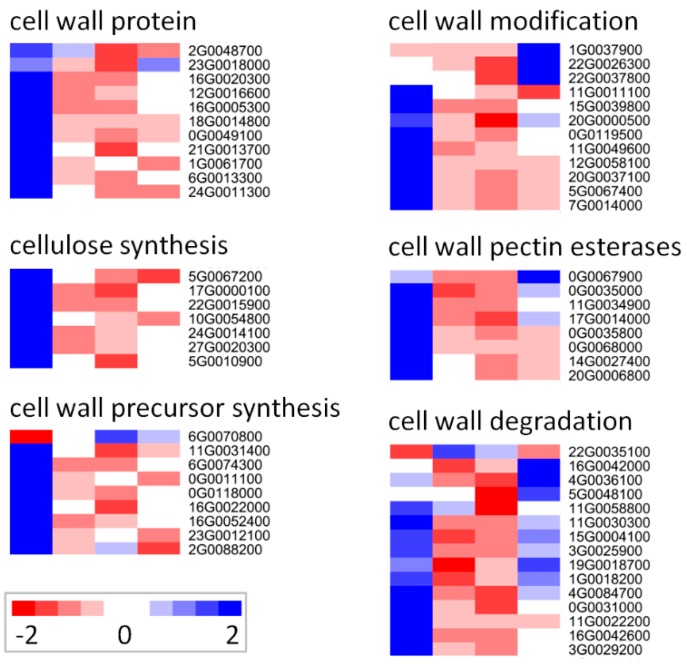
Expression profiles of DE genes related to cell wall. These genes are assigned into six subgroups of cell wall protein, cellulose synthesis, cell wall precursor synthesis, cell wall modification, cell wall pectin esterase, and cell wall degradation based on MapMan annotation. The FPKM values are transformed by 2-based logarithm and then normalized by row as scaled by the color bar. Each row indicates a single gene, and the cells (from left to right) represent the samples from 0, 6, 12, and 24 h after salt treatment, respectively.

In total, 62 DE genes were involved in cell wall pathways, including six subgroups of cell wall protein, cellulose synthesis, cell wall precursor synthesis, cell wall modification, cell wall pectin esterase, and cell wall degradation (Table S4). Amazingly, the expression levels of these genes from each subgroup were greatly downregulated by salt (Figure 6 and Table S4). The function of several genes has been well characterized in cell wall. For examples, spipo17g0000100 and spipo22g0015900 encoded homologs of cellulose synthase-like D3 (*CSLD3*) and *CSLD5*, respectively, mutants of the former initiated root hairs that burst at their tip soon after initiation in *Arabidopsis* [26], while mutants of the latter had reduced growth, reduced xylan level, and reduced xylan synthase activity in stems [27]; spipo27g0020300, spipo24g0014100, and spipo10g0054800 encoded homologs of *CESA1*, *CESA3*, and *CESA6*, respectively, much evidence suggested that *CESA3*, along with *CESA1* and *CESA6* were presented in the same plasma membrane complex for cellulose biosynthesis in *Arabidopsis*, and mutants of these three genes, respectively, exhibited cellulose defect in the primary cell wall [28,29]; spipo12g0016600 encoded a homolog of fasciclin-like arabinogalactan-protein 1 (*FLA1*), of which the mutants showed defects in shoot regeneration [30].

Besides, several cell wall-related genes were identified and participated in salt response. For examples, spipo0g0068000 is a homolog of a salt and chilling-sensitive gene *ATPMEPCRB* in *Arabidopsis*, spipo23g0018000 is a homolog of salt overly sensitive 5 (*SOS5*), of which the *Arabidopsis* mutants showed thinner cell walls, abnormal siliques, and inhibited root growth under salt stress [31].

Taken together, these results indicate that salt stress greatly inhibits the expression of genes involved in the biosynthesis and degradation pathways of cell wall.

### 2.6. Response of Abiotic Stress-Related Genes

In total, 32 DE genes referred to abiotic stress were identified responding to salt treatment. The GO categories of these DE genes are heat stress (19 genes), drought/salt stress (nine genes), cold stress (three genes), and touch/wounding (one gene, Table S5). 

These genes were grouped into three main clusters according to the hierarchical clustering (A1–A3, Figure S2). Overall, the expression of genes in cluster A1 (22%, 7 genes) was greatly downregulated from 6 h to 12 h but increased at 24 h. On the contrary, the genes in cluster A2 (25%, 8 genes) was greatly induced at 6 h but decreased from 12 h to 24 h. A homolog (Spipo9G0008100) of *ATJ3* that was involved in heat-stress but not in salt stress in *Arabidopsis* [32,33], was included in this cluster, suggesting functional diversification of *ATJ3*-like genes in different species. Compared to cluster A1 and A2, the genes in cluster A3 (53%, 17 genes) were expressed highest at 0 h, and their expression levels were dramatically downregulated from 6 h to 24 h. A homolog (Spipo23G0037000) of *ESK1*, which functioned as a negative regulator of cold stress [34], and a homolog (Spipo26G0006100) of *RD22*, which was mediated by abscisic acid (ABA) and responded to desiccation and salt stresses [35], were included in this cluster. Together, these results suggest different responses of abiotic stress genes under salt treatment.

### 2.7. Roles of Genes Related to Hormone Biosynthesis

In total, 61 hormone-related genes, referred to ABA, brassinosteroid (BR), auxin, cytokinin (CK), gibberellic acid (GA), ethylene, and jasmonic acid (JA), were differentially expressed under salt treatment. The hormone with the most abundant DE genes was auxin (20 genes), followed by BR (12), JA (9), ABA (9), and ethylene (7) (Table S7). Subsequently, the expression of genes related to the biosynthesis and degradation of each hormone was inspected, respectively. 

Nine-cis-epoxycarotenoid dioxygenase (NCED) is a crucial enzyme in the ABA biosynthetic pathway. The expression of a *NCED3*-like gene (Spipo5G0039400) was significantly increased from 6 h to 12 h and then declined at 24 h. Similar expression trends were observed in two *ABF1*-like genes (Spipo4G0008600 and Spipo7G0034500), which acts as an ABA-responsive element-binding factor to mediate the transcriptional regulation of ABA responses (Table S6).

IAA-amino acid conjugate hydrolase is a key enzyme to generate IAA from the hydrolysis of IAA–amino acid conjugates in auxin biosynthesis pathway. The expression of *ILL6*-like gene (Spipo4G0007100), which encodes an IAA-amino acid conjugate hydrolase, was slightly upregulated from 6 h to 12 h but downregulated at 24 h. However, *PIN1*, which encodes an auxin efflux carrier, together with dozens of auxin-responsive genes, were significantly downregulated from 6 h to 24 h after salt treatment (Table S6). These results indicate that auxin signal transduction pathway was weakened in *S. polyrhiza* upon salt stress.

Several critical genes involved in the BR biosynthesis pathway were found. Of which, the expression levels of *DWF7* and *BR6OX2* were upregulated, while genes with more abundant transcripts at normal conditions (Table S6), including *BAS1*, *HYD1*, *SMT1*, and *SMT2*-like genes were significantly downregulated in response to salt stress. In addition, many genes invovled in the signal transduction of BR were downregulated, such as *BRI1*, which encodes a leucine-rich repeat receptor kinase, brassinosteroid-responsive RING-H2 (Spipo11G0019400), meristem receptor-like kinase 2 (Spipo16G0046100), and EXORDIUM like 5 (Spipo17G0033100). These results indicated that the signal transduction pathway of BR was downregulated upon salt stress.

Gibberellin (GA) 3-oxidases catalyze the conversion of GA precursors to bioactive forms [36]. The expression of *GA3OX1*-like gene (Spipo21G0010100) was gradually increased from 0 h to 24 h in response to salt treatment. CYTOKININ OXIDASE/DEHYDROGENASE (CKX) encode cytokinin-degrading enzymes [37]. Two CKX-like genes (Spipo8G0036100 and Spipo1G0049000) were significantly downregulated under salt treatment. At the same time, the expression of a *ACO1*-like gene (Spipo23G0011700), which catalyzes the final step of ethylene biosynthesis, was greatly downregulated from 0 h to 24 h under salt treatment (Table S6). These results suggested that *S. polyrhiza* made efforts to increase GA and cytokinin activity but reduce ethylene level in response to salt stress.

It’s worthy to note that, the expression levels of three lipoxygenases (*LOX2*, *LOX3*, *LOX5*), an allene oxide synthase (*AOS*), an allene oxide cyclase (*AOC3*), and a 12-oxophytodienoate reductase (*OPR1*), which constitute a complete pathway of JA biosynthesis, were all significantly downregulated by salt stress. Coincidently, the expression of *JAZ1*, which is involved in JA signaling and transduction, was also greatly downregulated (Table S6).

Taken together, these results suggest diverse regulation of hormone signal transduction responding to salt stress, e.g., the expression of genes related to ABA, CK, and GA biosynthesis and signal transduction was greatly upregulated, while the expression of genes related to IAA, BR, ethylene, and JA biosynthesis and signal transduction was significantly downregulated upon salt treatment.

### 2.8. Roles of Transcription Factor (TF)

In total, 106 TF genes representing 18 families were differentially expressed responding to salt stress. The four most abundant TF families were bHLH (13), C2H2 (11), HB (11), and MYB (11), followed by AP2/EREBP (10), and bZIP (7, Table S7). 

According to the expression patterns (Figure 3A), tens of TFs were respectively classified in group G1 to G6 (Table S7). The groups with abundant TF members were G6 (27 genes), G2 (24), and G5 (23). In group G6, several abiotic stress-related TFs, including *MYB96* for drought and ABA signaling and *RAV1* for cold stress, were included. Many TFs related to leaf initiation and differentiation (*TB1*, *TCP4*, and *ZFP1*), cell differentiation (*SCAP1*), trichome growth (*HDG2*), and auxin response (*ARF19*) were also included in Group G6. Group G2 contained also several abiotic stress-related TFs, including *CBF2* for cold and ABA, *ERF53* for drought, and *STZ* for salt stress, in accordance with the functional category enrichment of this group (Figure 3). However, only a few TFs related to auxin response (*SHY2* and *AUX2-11*), cell differentiation (*PDF2*), and trichome differentiation (*ZFP8* and *NOK*) were included in group G5. 

The groups with less abundant TF members were G4 (17 genes), G3 (9), and G1 (6). Several hormone-related TF members, including *ATH1* for GA biosynthesis regulation and *ETT* for auxin stimulus, were included in group G3. In comparison, several salt-responsive TF members, including *MYB113*, *WRKY33*, and *STZ*, were included in group G1 and G4, respectively.

Taken together, these results suggest that the TF members involved in abiotic stress, cell differentiation, and hormone biosynthesis and signaling responded to salt treatment in diverse expression patterns.

### 2.9. Responses of Cation Transporters That Maintain Ion Homeostasis during Salt Stress

**Figure 7:**
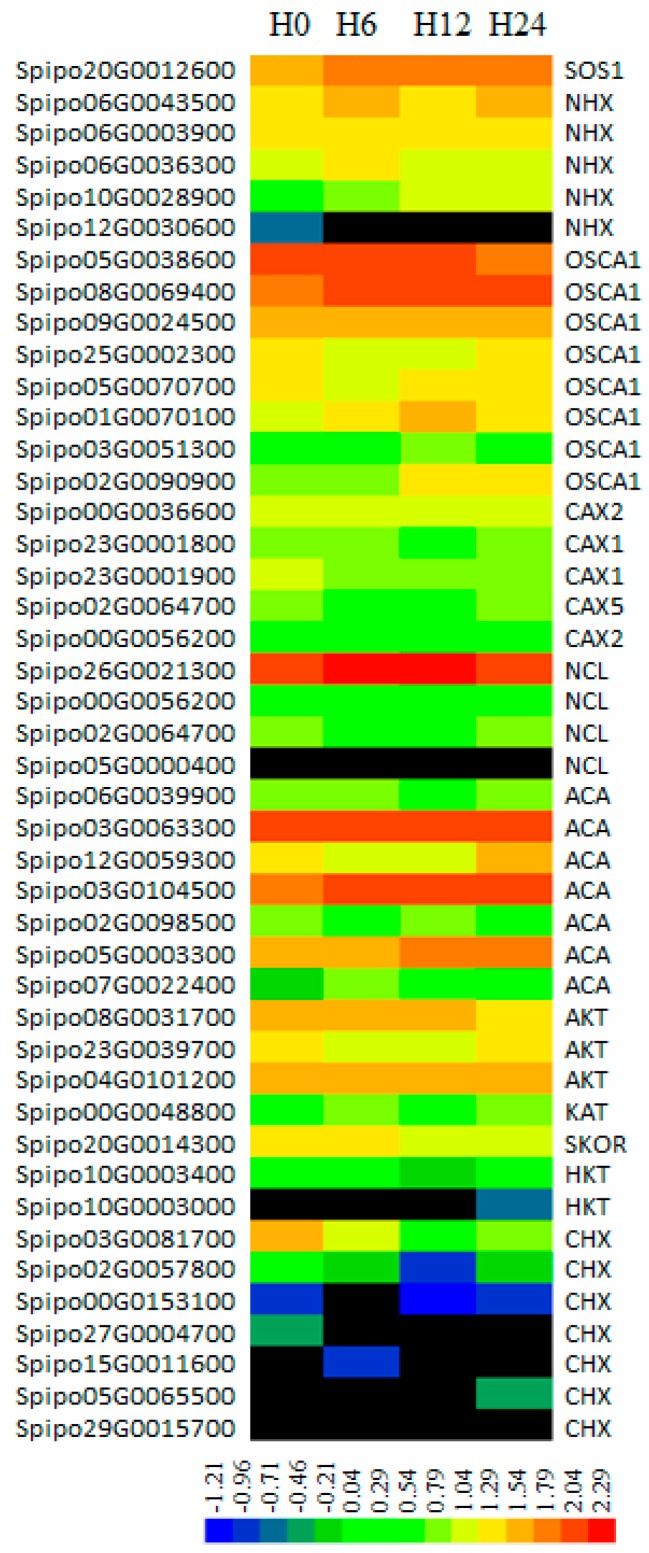
Expression pattern of cation transporters. The accession numbers are provided on the left column, and the gene identities are provided on the right column. The FPKM values of each gene at 0, 6, 12, and 24 h after 100 mM NaCl treatment, respectively, were transformed by 10-based logarithm and are presented in color as scaled by the color bar.

During salt stress excessive Na^+^ taken by the cell must be extruded from the cytoplasm to reduce toxicity [38], the detoxification mechanism includes sequestration of Na^+^ to the vacuole by Na^+^/H^+^ antiporters at the tonoplast (NHX) and extrusion of Na^+^ outside of the plasma membrane by SOS1 transporters [39,40]. One *SOS1* gene and five *NHX* genes were identified in the genome of *S. polyrhiza*, they were mostly more abundant upon salt stress except Spipo12G0030600, which was weakly expressed in stress-free medium but downregulated to an undetectable level under salt stress (Figure 7). However, none of these changes were statistically significant, which may explain the 16.4-fold increase in Na^+^ content in the fronds of *S. polyrhiza* at 24 h of salt stress.

Osmosensing calcium antiporters (OSCA) play key roles in salt stress signaling [41]. A total of eight *OSCA* genes were identified in *S. polyrhiza* genome. Three of them (Spipo5G0038600, Spipo25G0002300, and Spipo5G007070) including the most predominantly expressed gene Spipo5G0038600 were downregulated at one or more time points, while the other *OSCA* genes were slightly upregulated. Five Ca^2+^/H^+^ antiporter genes (*CAX*) and four Na^+^/Ca^2+^ exchanger genes (*NCL*) were also identified, Spipo26G0021300 (*SpNCL1*) was dominant in transcripts among these genes, and it was only slightly upregulated under salt stress (Figure 7). This result is different compared to that in *Arabidopsis*, in which the broadly expressed *AtNCL* was strongly stimulated by abiotic stresses [42]. The expression changes of other calcium transporting genes were not obvious. 

Most members of the Shaker family inward rectifying potassium channel, including AKT (Spipo8G0031700 and Spipo23G0039700), SKOR (Spipo20G0014300), and HKT (high-affinity K^+^ transporter, Spipo10G0003400), and a CHX K^+^ antiporter gene (Spipo03G0081700), were downregulated at one to three time-points after salt stress (Figure 7). At the same time, a KAT member gene (Spipo0G0048800) that encodes a guard cell outward K^+^ channel protein was upregulated at 6 h after salt treatment, although it was not statistically significant (*p* < 0.05). These results may explain partly the decrease of potassium content in the plants treated with 100 mM NaCl. 

### 2.10. Quantitative RT-PCR Validation of DE Genes

**Figure 8:**
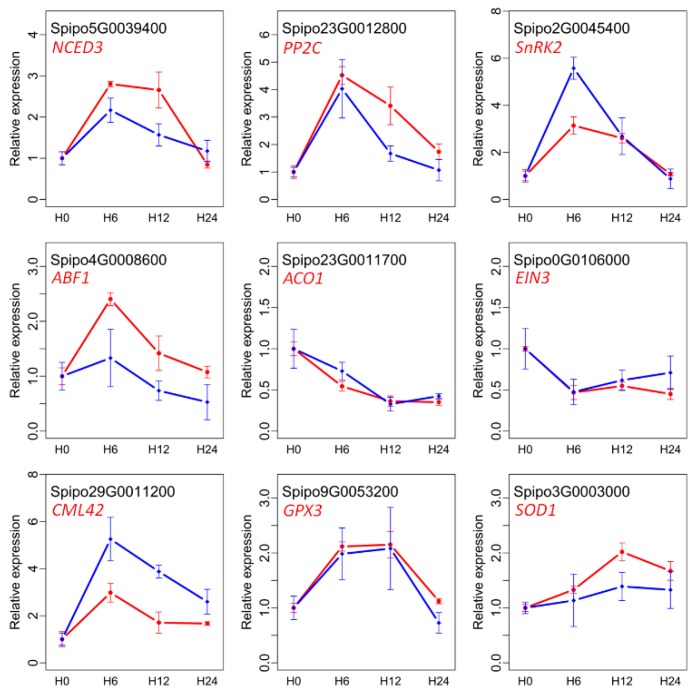
Validation of gene expression patterns using qRT-PCR. Values are presented as mean ± SD of three independent replicates. Blue and red lines represent the expression values from qRT-PCR and RNA-seq, respectively.

To verify the transcriptome results, in total nine genes, including *CML42* involved in Ca^2+^ signaling, *GPX3* and *SOD1* involved in ROS-scavenging, *NCED3*, *PP2C*, *SnRK2*, and *ACO1* related to hormones, and *ABF1* and *EIN3* related to TFs, were selected to perform qRT-PCR (Figure 8 and Table S9). The results revealed that the expression trends were consistent (*R* = 0.76–0.98) between these two independent measurements.

## 3. Discussion

### 3.1. Physiological Performance and Maintenance of Ion Homeostasis in S. Polyrhiza under Salt Stress

Duckweed has great potential in modern agriculture and industry, mainly due to its great abilities for municipal and industrial wastewater treatment (e.g., remove nutrients or heavy metals) and its good characteristics (e.g., fast growth and high starch content) for bio-ethanol production. Therefore, in the past decades, many studies have focused on the physiological performances of duckweed in response to diverse treatments such as nutrient starvation, NH_4_^+^ toxicity, metal pollution, and salt stress [5,15,20,43]. Under nutrient starvation conditions, starch content and the AGPase activity were significantly increased, while the growth rate, protein content, and photosynthesis and respiration rate were dramatically inhibited [15,44]. NH_4_^+^ toxicity also significantly inhibited the growth of *L. minor*, however, the MDA content and the activities of ROS enzymes such as SOD and POD were greatly increased [20]. Under high Co^2+^ and Ni^2+^ concentration (e.g., 5 mg L^-1^), the growth rate, net photosynthesis rate, chlorophyll content, and Rubisco activity were significantly inhibited, while the starch content was increased sharply attributed to the increase in AGPase and soluble starch synthase activities and the decrease in α-amylase activity [43]. 

Duckweeds are sensitive to salinity stress. Cheng et al. (2011) performed physiological analysis of *S. polyrhiza* strain DR (a local strain isolated from a creek in the Taiwan Island) under 100, 150, and 200 mM NaCl stress, and found that the growth of duckweed was significantly reduced in a dose-dependent manner. The relative fresh weights were reduced by 24% and 37% after cultured for 4 days under 100 mM and 200 mM salt stress, respectively [5]. Some fronds started discoloration two days after 150 mM and 200 mM NaCl treatments [5]. However, the content of photosynthetic pigment was not decreased at a statistically significant level until 4 days of treatment [5]. The same research group [6] further studied oxidative stress of *S. polyrhiza* strain DR under long-term (6 and 12 days) 200 mM NaCl stress, and found that plants showed chlorotic effect after 4–6 days, and chlorophyls were reduced significantly after 6 days of treatment. Liu et al. (2017) studied the effect of salt stress on the removal of N and P by common duckweed, *L. minor*, and found that salt stress exerted injury to duckweed and reduced N and P removal, and severe salt stress (100 mM NaCl) even induced duckweed to release N and P to water [7]. Therefore, when *L. minor* is used to remove N and P from wastewater, salinities below 75 mM NaCl is required [7]. Salt stress also inhibited the removal of boron from water by *L. minor*, and NaCl salinity below 100 mM is suggested when *L. minor* is used in boron removal from wastewater [45]. Sree et al. (2015) compared salt stress tolerance of 34 strains of duckweeds from across all 5 genera and 13 species, and found large interspecific and intraspecific variations in salt tolerance [9]. *S. polyrhiza* strain 7160 showed the highest tolerance among the tested strains, and the NaCl concentration that reduced its growth by 50% was caculated to be 377 mM, while *Wolffia globosa* strain 9667 was the least tolerant strain and its NaCl concentration for 50% growth reduction was only 10.1 mM [9]. Overall, *S. polyrhiza* strains mostly are more tolerant to salt stress than other duckweed species, although there existed intraspecific variations [9]. Our results also showed that *S. polyrhiza* 7498 grew well under 50 mM and 100 mM NaCl condition for about 4 days, and the fresh weights were only reduced by 7.4%, 8.2%, and 22.2% in the first day for N50, N100, and N150, respectively, and were respectively reduced by 26.3%, 58.8%, and 69.7% by the end of the experiment (Day 6, Figure 1A). Discoloration only occurred after five days of culture at high NaCl concentrations (100 mM and 150 mM), and many fronds of N150 turned yellow at Day 7 (Supplementary Figure S1), which is similar to previous reports [5,6].

Besides growth reduction, high salt concentration (e.g., 200 mM NaCl) also reduced photosynthetic pigment content, but significantly enhanced MDA content and increased the activities of several ROS enzymes including POD, SOD, APX, and CAT [5,6]. However, information is limited regarding the changes of Rubisco activity, AGPase activity, starch content and ion contents in duckweed under salt stress. 

In a previous report [7], removal of phosphorus and nitrogen from water by duckweed was strongly inhibited by salt stress, and duckweed even released N and P to water under high stress. Our research also indicates that ion intake and homeostasis of *S. polyrhiza* was strongly disturbed by salt stress, and plants accumulated a significant amount of sodium ion, which was 16.4 times the normal content. At the same time, potassium and calcium contents were greatly decreased (Figure 1), similar to the reduction of phosphorus and nitrogen intake in *L. minor* under salt stress [7]. These results suggested that the sodium detoxification mechanism in *S. polyrhiza* is inefficient. In many plants, hyperosmotic stress triggers opening of the osmosensitive channels, leading to a rapid downstream signaling cascade [46]. *AtOSCA1*, known as a hyperosmolality-gated calcium-permeable channel, mediates Ca^2+^ increase and is vital for osmosensing in *Arabidopsis* [41]. Eight *OSCA* genes were identified in *S. polyrhiza* in this research, however, they were mainly downregulated upon salt stress. Some of the sodium antiporter genes, e.g., *SOS1* and *NHXs*, were slightly upregulated (Figure 7). However, in salt tolerant plants, for example, *Kochia scoparia* (L.) Schrad., its *SOS1* gene expression was upregulated by 1.5 and 2.5 times, while its *NHX* gene was upregulated by one and two times when treated with 150 and 300 mM NaCl, respectively [47]. Similarly, *SOS1* genes in salinity tolerant *Chrysanthemum species* was more strongly induced by salt stress than those carried by the non-tolerant ones [48], and stress induced a higher accumulation of Na^+^ and more reduction of K^+^ in the stress-sensitive species *C. morifolium* [48]. Through these species comparisons, we conclude that the Na^+^ detoxification in *S. polyrhiza* is not efficient.

We found great reduction in calcium concentration in *S. polyrhiza* during salt stress (Figure 1). However, many calcium transporter genes including *CAX*, *NCL*, and *ACA* genes were not significantly changed at the transcriptional level (Figure 7). CAXs are tonoplast Ca^2+^/H^+^ antiporters that mediate the sequestration of Ca^2+^ from the cytosol, usually into the vacuole [49]. Overexpression of a chimeric *Arabidopsis* *CAX2B* in potato tubers increased calcium Ca^2+^ content by 50-65% compared to wild-type tubers [50]. In this research, five CAX genes were identified, four of them (Spipo23G0001800, Spipo23G0001900, Spipo02G0064700, and Spipo00G0056200) were slightly downregulated at one or more time points (Figure 7), which may partially explain the reduction of calcium content in *S. polyrhiza* under salt stress (Figure 1). NCLs are Na^+^/Ca^2+^ exchangers in the plasma membrane, *AtNCL* in *Arabidopsis* is stimulated by abiotic stress [42]; Higher expression of *NCL* in the root of soybean resulted in lower accumulations of Na^+^, K^+^, and Cl^-^ in the shoot under salt stress, and improved soybean grain yield by 3.6–5.5 times in saline field conditions [51]. In this research, four NCLs were identified in *S. polyrhiza*, but none of them were upregulated, while one of them (Spipo02G0064700) was even slightly downregulated (Figure 7). 

We also found great reduction in potassium content under salt stress (Figure 1). In higher plants, the Shaker family potassium channels have been shown to play crucial roles in the uptake of K^+^ from soil and subsequent allocation of this ion within the plant [52]. Yeast and rice cells overexpressing *OsKAT1* showed enhanced salt tolerance, increased cellular K^+^ content and reduced Na^+^ compared with the non-transgenic lines under salt stress [53]. A shaker family member *AtKC1* were strongly induced by salt stress in *Arabidopsis* [54]. CHX genes belong to the cation:proton antiporter-2 (CPA2) family (or CHX family), and were predicted to encode Na^+^, K^+^/H^+^ antiporters [55]. A member of this family, *AtCHX17*, was strongly induced by salt stress and potassium starvation, and the knockout mutants accumulated less K^+^ in roots in response to salt stress and potassium starvation compared with the wild type [56]. In this research, the performance of the Shaker family proteins in *S. polyrhiza* under salt stress were investigated. Results indicated that an AKT gene (Spipo8G0031700) and a CHX gene (Spipo3G0081700) were significantly downregulated, while most of the rest potassium transporter genes including CHX, SKOR, and HKT genes were slightly downregulated (Figure 7). These results indicated that the performance of potassium transporter genes in *S. polyrhiza* is quite different compared to other plants, and may have resulted in the great reduction of K^+^ content under salt stress.

Due to the disturbance of ion homeostasis under salt stress, Rubisco activity and AGPase activity of *S. polyrhiza* were decreased in the first day of salt stress, but increased gradually in the following days, and accordingly, the starch content was decreased in the first day, but increased gradually in the following days. The starch content was even higher than the control from Day 3 to Day 5 in the 150 mM treatment (Figure 1), partly in agreement with a former research in duckweeds, in which many duckweed species showed increased starch content at the end of salt treatment [9]. A similar result was also found in other plants, e.g., *Piptadenia moniliformis* Benth., however, starch content was only increased in the roots of the seedlings under 100 mM NaCl, probably in response to the toxic effects of Na^+^ [57]. In *Anabaena doliolum*, rubisco activity was also increased by salt treatment, at the same time, carbon fixation was inhibited and glycolate metabolism was enhanced, suggesting that rubisco may function as oxygenase [58]. In *Kalidium foliatum* (Pall.) Moq., rubisco activity was decreased at low NaCl concentrations (100 and 250 mM) but incrased at high concentrations (400 and 500 mM) [59]. This dose-dependent change pattern of rubisco activity is similar to the temporal-dependent changes of rubisco activity in our research. Referring to the published data, the increase of Rubisco and AGPase activities and starch contents at late stage of salt stress in *S. polyrhiza* (Figure 1) was not necessarily resulted from enhanced photosynthesis, alternatively, upregulated rubisco may just play as oxygenase, and carbon source for starch accumulation may be derived from alternative source, e.g., inhibited cell wall biosynthesis and plant growth (Figure 6), which may represent pre-dormancy reactions for escaping from salt stress, since plants usually accumulate starch before dormancy [60,61].

### 3.2. Expression of Genes Related to Cell Wall, Photosynthesis, and Carbohydrate Metabolism

Many studies have observed that salt stress significantly reduced vegetable growth, inhibited photosystems I and II activities, and influenced starch accumulation in duckweed, and the effects were concentration-dependent under salt condition [7,8,9]. However, less information is available regarding the expression changes of genes involved in these pathways. 

In other plants, it has been well demonstrated that the expression of cell wall-related genes was significantly downregulated under salt condition [62,63]. Accordingly, in our study, we found that as many as 62 DE genes involved in cell wall pathways, including six subgroups of cell wall protein, cellulose synthesis, cell wall precursor synthesis, cell wall modification, cell wall pectin esterase, and cell wall degradation, were dramatically downregulated by salt (Figure 6), which might explain why the growth of duckweed was greatly inhibited under salt stress. 

In our study, we found that the expression levels of genes involved in PSI, PSII, and their related polypeptide subunits were dramatically downregulated by salt stress. Besides, several genes related to electron carrier and state transition of light reaction were also downregulated, in agreement with a recent report that salt stress caused a significant inhibition of both PSII and PSI electron transport activities and a decrease in the number of active PSII reaction centers [8]. We also found that one chlorophyll synthase (spipo23G0037100) and two protochlorophyllide reductase (spipo16G0033900 and spipo1G0039600), which located on the pathways of chlorophyll biosynthesis, were significantly downregulated by salt stress, indicating that these are the crucial genes responsible for the reduction of photosynthetic pigments under salt stress [5,6].

Besides photosynthesis, the genes involved in sucrose and starch metabolism were significantly enriched in plants under salt stress [64,65]. This conclusion was also supported by our study since the genes related to the degradation of major CHO metabolism were significantly enriched (Figure 3). Further inspection revealed that the expression of ADP-glucose pyrophosphorylase (*APL1*) which catalyzes the first and rate-limiting step of starch biosynthesis was significantly decreased; while many genes located on the starch-degradation pathway, including cytosolic/plastidic alpha-glucan phosphorylase, alpha- and beta-amylase, glucan water dikinase (*SEX1*), and disproportionating enzyme 2 (*DPE2*), were greatly induced by salt stress. These results explained the decrease of AGPase activity and starch content of *S. polyrhiza* within 24 h under salt stress in our study, however, since gene expression profiles during late stage of salt stress were not investigated, the increase of AGPase activity and starch content at late stage (e.g., 120 h) of salt stress (Figure 1) remains to be illustrated. 

### 3.3. Expression of Genes Related to Redox and Ca^2+^ Signaling

Salt stress usually induces rapid accumulation of ROS, which causes oxidative stress-induced toxic effects in plants [5,6]. The elevated ROS must be regulated at suitable levels in plant cells, thus ROS can act as important signaling molecules contributing to stress injury, as transgenic plants over-expressing ROS scavengers or mutants with higher ROS-scavenging ability exhibit enhanced tolerance to various environmental stimuli [66]. Under salt stress condition, MDA and H_2_O_2_ content were significantly increased, and accordingly, the activities of several ROS-scavenging enzymes including SOD, APX, CAT, POD, and GR were greatly increased in *S. polyrhiza* [5,6]. In our study, a total of 17 DE genes related to redox metabolism were identified (Table S1). Of which, glutathione peroxidase 3 (*GPX3*), which functions as both a redox transducer and a scavenger in ABA and abiotic stress response [67], and superoxide dismutase 1 (*SOD1*), which is involved in salt stress response and superoxide radicals removal [68], were greatly induced by salt, suggesting that these two genes are key regulators of ROS-scavenging in *S. polyrhiza* under salt stress. 

In addition to ROS, the calcium ion (Ca^2+^) is also recognized as a secondary messenger and plays a key role in signal transduction under salt condition [69]. In *Arabidopsis*, plants over-expressing *AtCPK3* and *AtCPK6* showed enhanced tolerance to salt, while *atcpk6* mutant exhibited no obvious phenotypes [70,71]. *AtCML9* was also found to be involved in salt stress tolerance through its effects on the ABA mediated pathways [72]. In *S. polyrhiza*, addition of CaCl_2_ to the salt stressed plants increased the contents of proline, and lowered the degree of injury caused by NaCl [73], indicating the critical involvement of Ca^2+^ signaling in response to salinity treatment. In this study, a total of 19 DE genes involved in Ca^2+^ signaling were found (Table S1). Of which, *CML42* was greatly induced while *CAM5*, *CPK1*, and *CPK33* were significantly downregulated upon salt treatment. It is worthy to note that one gene encoding *P5CS1* (spipo6g0074800), which is a crucial enzyme in proline biosynthesis, was dramatically increased under salt stress, while another gene encoding *GS1* (spipo15G0035800) was greatly decreased in response to salt treatment, in accordance with the result of a previous report [73]. 

Together, these results suggest that the genes related to redox and Ca^2+^ signaling played a crucial role in response to salt stress in *S. polyrhiza*. 

### 3.4. Regulatory Networks of Salt Stress in S. Polyrhiza

**Figure 9:**
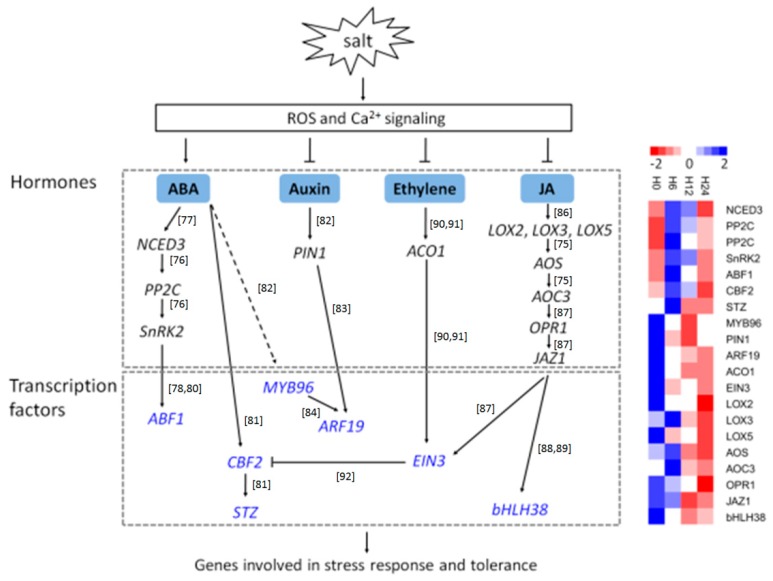
Regulatory networks underlying salt stress in duckweed (*Spirodela polyrhiza*). Solid lines indicate interactions conincident with previous studies, while dashed lines indicate interactions already reported but not identified in the present work. Normal arrows indicate positive regulation, while the T-bars represent negative (inhibitory) regulation. Transcription factors are highlighted in blue. Heatmaps of all genes in the networks are presented on the right.

Following the genes involved in signals perception (such as ROS and Ca^2+^ signaling), hormone and TF genes are also recognized as important players involved in salt stress [74,75]. Subsequently, these DE genes were assigned into relevant regulatory networks according to the MapMan annotation as well as their co-expression trends, to explore the common and different mechanisms underlying the salt stress response between *S. polyrhiza* and other plants (Figure 9). 

ABA is a key hormone with important roles in various plant stresses [76]. Therefore, it is critical to understand the expression changes of genes involved in ABA signaling pathways. The expression of *NCED3*, which is a crucial gene of ABA biosynthetic pathway, is dramatically induced by dehydration and high salinity [77]. Consistently, in this study, we found that *NCED3* of *S. polyrhiza* was strongly upregulated by salt stress especially during the early period (e.g., from 0 h to 12 h). In addition, one *SnRK2* and two *PP2C* genes, which respectively act as positive and negative regulators of ABA signaling in crop plants [76], all exhibited similar expression patterns as that of *NCED3*, indicating that the two *PP2C* genes in *S. polyrhiza* might function differently compared with their homologs in other plants. ABFs are the bZIP family members that bind to ABA-responsive elements (ABREs) and regulate gene expression [78], while ABA-activated *SnRK2* kinases are responsible for ABA-dependent phosphorylation of ABFs [79,80]. Therefore, it is reasonable that *ABF1* and *SnRK2* in *S. polyrhiza* showed similar expression patterns upon salt stress, and they were both upregulated at 6 h, but return to the normal level at 12 h (Figure 9), in accordance with the proposal that ABA-activated *SnRK2s* play essential roles in regulating the expression of ABA-responsive genes through phosphorylating AREBs/ABFs [76]. Two other genes, *STZ* and *CBF2*, which were strongly induced by cold and salt stress in *Arabidopsis* [81], also exhibited ABA-induced expression pattern as *NCED3*, indicating that these two genes were also involved in the ABA-mediated signaling pathway under salt stress in *S. polyrhiza*. 

Auxin also plays important roles in salt stress response, since endogenous IAA contents in leaves and roots were significantly decreased in plants under salinity condition [82,83]. *MYB96* is a key TF and regulates drought stress response by integrating ABA signals into an auxin signaling pathway in *Arabidopsis* [84]. An *Arabidopsis* mutant overexpressing MYB96 exhibited enhanced drought resistance [84]. However, the expresssion pattern of *MYB96* in *S. polyrhiza* was downregulated upon salt stress (Figure 9), obviously distinct from *NCED3* in the ABA signal pathway, but similar to that of an auxin efflux carrier *PIN1*, an auxin response factor *ARF19*, and several auxin-responsive *GH3* genes (Figure 9), suggesting that *MYB96* in *S. polyrhiza* might be only involved in the auxin signals and does not act as a molecular link to mediate the ABA-auxin cross-talk in salt stress response in *S. polyrhiza*.

In addition, ethylene and JA were demonstrated to play essential roles in regulating plant response to salt stress [75]. Transgenic expression of *TaAOC1* gene, encoding an allene oxide cyclase (AOC), increased JA levels and enhanced salt tolerance in *Arabidopsis*, suggesting that JAs positively regulate salt tolerance [85,86]. However, in our study, *ACO1* in *S. polyrhiza* was downregulated by salt stress (Figure 9). It is also notable that salt stress strongly downregulated the expression of three lipoxygenases (*LOX2*, *LOX3*, *LOX5*), one each of allene oxide synthase (*AOS*), allene oxide cyclase (*AOC3*), and 12-oxophytodienoate reductase (*OPR1*) that located on the JA biosynthesis pathway [87]. As a key player involved in JA signaling and transduction, JAZ protein interacted with *EIN3* and a few bHLH TFs under drought and salinity conditions [75,88,89]. Consistently, the expression of *JAZ1*, *EIN3*, and *bHLH38* was greatly downregulated upon salt stress. These results suggested that JAs act as negative regulators of salt tolerance in *S. polyrhiza*. Under salinity stress condition, the expression levels of lots of ethylene-responsive genes were significantly altered [90]. It seems that ethylene negatively affected salt tolerance because a correlation was observed between the increased ACC levels and reduced salt tolerance in *Arabidopsis* [91]. In addition, plants over-expressing *TaACO1*, which catalyzes the final step of ethylene biosynthesis, exhibited enhanced ethylene accumulation and increased salt sensitivity, and reduced the expression of two key TFs, *DREB1B*/*CBF1* and *DREB1A*/*CBF3* [75,92]. As expected, the expression of *ACO1*, together with *EIN3*, which acts as a positive regulator of the ethylene signaling and a negative regulator of the CBF pathway [93], was strongly downregulated upon salt stress.

## 4. Methods

### 4.1. Plant Materials and Salt Treatment

*S. polyrhiza* (strain No. 7498) was selected and cultured at the Biological Collection Centre of the Institute of Tropical Bioscience and Biotechnology, Chinese Academy of Tropical Agricultural Sciences, Hainan, China. To reveal the physiological changes of duckweed responding to salt stress, 0.5 g three-frond colonies were inoculated with three replicates in a 250 mL flask containing 100 mL MH medium supplemented with 0, 50, 100, and 150 mM NaCl, respectively, and cultivated under 16 h daily photoperiod at 40 μmol m^−2^ s^−1^ photosynthetic active radiation (PAR) at 25 ± 1 °C as previously described [94]. 

*S. polyrhiza* samples were respectively collected at a total of ten time-points including 0, 3, 6, 12, 24, 48, 72, 96, 120, and 144 h after salt treatment, to survey the physiological traits including fresh weight, Rubisco activity, AGPase activity, and starch content, and each was repeated three times.

Rubisco activity, AGPase activity, and starch content were measured spectrophotometrically, respectively. Briefly, 0.25 g samples were ground and homogenized in 2 mL 0.01 M PBS extraction buffer (pH7.8), then the homogenate was centrifuged for 15 min at 4000 rpm at 4 °C. The resulting supernatant was collected to determine the Rubisco activity, AGPase activity, and starch content, respectively, by using plant enzyme-linked immunosorbent assay (ELISA) kits (Jianglai Biotechnology, Shanghai, China).

To investigate the ionic status during salt stress, *S. polyrhiza* 7498 was cultured as described above, and 100 mM NaCl was supplemented in the MH medium. Samples were collected with six replicates at 0, 6, 12, 24, and 72 h after treatment, respectively. These samples were washed in deionized water briefly, and ground into powder in liquid nitrogen, and freeze-dried for 24 h. The contents of sodium and potassium ions were measured with a flame photometer (Sherwood, Model 410, UK), and the calcium ion was measured with an atomic absorption spectrometer (PerkinElmer, Model PinAAcle 900T, USA). 

To investigate the transcriptome changes of duckweed in response to salt stress, *S. polyrhiza* 7498 was cultured as described above. Samples were collected with three replicates at 0, 6, 12, and 24 h after 100 mM NaCl treatment, respectively. These samples were immediately frozen in liquid nitrogen and stored at −80 °C for RNA-seq sequencing.

### 4.2. RNA Extraction and Sequencing 

The total RNA extraction, transcriptome libraries preparation, and RNA sequencing were conducted by the Annoroad Gene Technology Corporation (Beijing, China). Briefly, total RNA was extracted for each sample using RNA plant reagent kits (Tiangen, China) [11]. The integrity and quality of total RNA were checked by ND-2000 spectrophotometer (Thermo Scientific Inc., USA) together with Agilent 2100 Bioanalyzer (Agilent, USA). The RNA-seq libraries were produced using Illumina TruSeq^TM^ RNA sample prep Kit (Illumina, San Diego, CA, USA) with Ribo-Zero Magnetic kit for rRNA removal following the manufacturer’s recommendations, and sequenced using Illumina Hiseq 4000 in PE150 model.

### 4.3. RNA-Seq Data Analysis

As previously described [10,11], adapters were trimmed from raw reads using FASTX-toolkit pipeline (http://hannonlab.cshl.edu/fastx_toolkit/). The quality of sequence was checked using FastQC (http://www.bioinformatics.babraham.ac.uk/projects/fastqc/), and low-quality reads were removed using customized Perl scripts. Clean reads were aligned to *S. polyrhiza* genome (version 2) derived from phytozome database (https://phytozome.jgi.doe.gov/pz/portal.html#!bulk?org=Org_Spolyrhiza) using Tophat v2.0.10 (http://tophat.cbcb.umd.edu/) [95]. Raw count were generated by Cuffdiff embedded in Cufflinks software v2.1.1 [96], and subsequently normalized by library sizes using edgeR [97]. Differential expressed (DE) genes were identified by DESeq [98] based on pair-wise comparisons setting FDR < 0.05 and |log_2_FC| > 1. Gene expression levels were normalized as Fragments Per Kilobase of exon per Million fragments mapped (FPKM). Significance of expression level differences of certain genes was evaluated by ANOVA at a significance level of 0.05.

### 4.4. Clustering and Functional Enrichment

Duckweed genes were functionally annotated and assigned into different hierarchical categories using MapMan software [99]. Significantly enriched functional categories were determined by Fisher’s exact test as previously described [10,11]. To explore the dynamic expression patterns of genes in response to salt stress, DE genes were assigned into different groups by hierarchical clustering with Pearson correlation method embedded in MEV [100], and the number of groups was determined using the Figures of Merit (FOM) method. MEV program was also applied for the heatmap visualization of gene expression.

### 4.5. Quantitative RT-PCR Analysis

To validate the results of RNA-seq, a total of nine DE genes, related to Ca^2+^ signaling, ROS-scavenging, hormone metabolism, and transcription factor, were picked and analyzed by qRT-PCR (Table S9). The 18S rRNA gene was used as an endogenous control. The qRT-PCR was performed on a Stratagene Mx3000P instrument (Stratagene, CA, USA) using SYBR-green (TaKaRa, Dalian, China), and the procedures were as follows: 30 s at 95 °C; followed by 40 cycles of 10 s at 95 °C and 30 s at 60 °C. Finally, a thermal denaturing step was conducted to generate the melt curves for amplification specificity verification. Each sample was measured with three biological and two technical replicates, and the relative expression levels were calculated using the 2^−ΔΔCt^ method. Pearson correlation was used to calculate the correlation coefficient between qRT-PCR and RNA-seq results. 

## Supplementary Materials

The following are available online at https://www.mdpi.com/2073-4425/10/10/743/s1, Figure S1. Performances of *S. polyrhiza* L. inoculated on medium conatining 50, 100, and 150 mM NaCl. Days after treatments are indicated on the right of each photos. Red arrows indicated discolorated plants. Figure S2. Heatmap of genes related to abiotic stress. These genes were grouped into three main clusters (A1-A3) according to their expression patterns. Blue and red cells (from left to right) represent log2-transformed FPKM values from 0, 6, 12, and 24 h after salt treatment, respectively. Table S1. Summary of 2156 DE genes identified in response to salt stress. Table S2. Summary of DE genes related to photosynthesis. Table S3. Summary of DE genes related to sucrose and starch biosynthesis -degradation pathways. Table S4. Summary of DE genes related to cell wall. Table S5. Summary of DE genes related to abiotic stress. Table S6. A list of 61 hormone related DE genes in this study. Table S7. A list of 106 differentially expressed TFs. Table S8. Species and strains used in salt stress related papers. Table S9. qRT-PCR results and the primers used in this study.
