# Supplementary figures and images for: Physiological and Transcriptomic Analysis Reveals Distorted Ion Homeostasis and Responses in the Freshwater Plant Spirodela polyrhiza L. under Salt Stress
Source: Genes (Basel). 2019 Sep 24;10(10):743. doi: 10.3390/genes10100743 (PMC6826491; doi:10.3390/genes10100743)

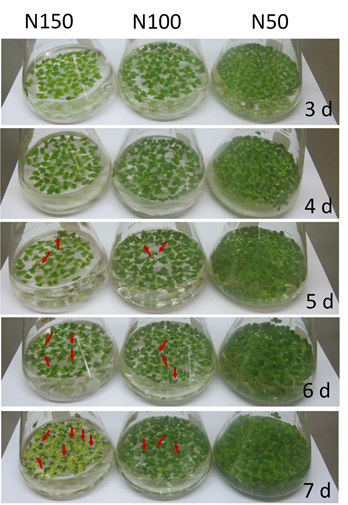

Supplement: Supplementary file 1 [file genes-10-00743-s001.zip › Supp_Figure_Tables/Fig.S1.jpg]

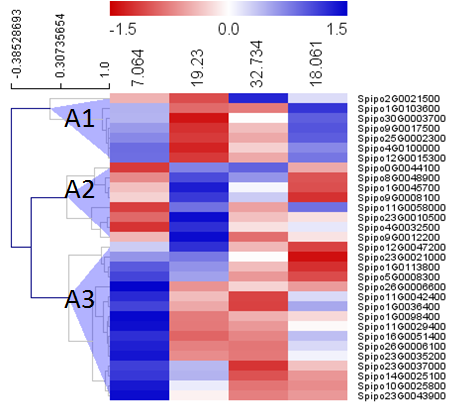

Supplement: Supplementary file 1 [file genes-10-00743-s001.zip › Supp_Figure_Tables/Fig.S2.tif]
